# Edge artificial intelligence wireless video capsule endoscopy

**DOI:** 10.1038/s41598-022-17502-7

**Published:** 2022-08-12

**Authors:** A. Sahafi, Y. Wang, C. L. M. Rasmussen, P. Bollen, G. Baatrup, V. Blanes-Vidal, J. Herp, E. S. Nadimi

**Affiliations:** 1 Applied AI and Data Science (AID), Maersk Mc-Kinney Moller Institute, Faculty of Engineering, University of Southern Denmark, Odense, Denmark; 2 Biomedical Laboratory, Department of Clinical Research, Faculty of Health Sciences, University of Southern Denmark, Odense, Denmark; 3 Department of Surgery, Odense University Hospital, Odense, Denmark; 4 Department of Clinical Research, University of Southern Denmark, Odense, Denmark; 5 Danish Center for Clinical Artificial Intelligence (CAI-X), Odense, Denmark

**Keywords:** Cancer, Gastroenterology, Engineering

## Abstract

Gastrointestinal (GI) tract diseases are responsible for substantial morbidity and mortality worldwide, including colorectal cancer, which has shown a rising incidence among adults younger than 50. Although this could be alleviated by regular screening, only a small percentage of those at risk are screened comprehensively, due to shortcomings in accuracy and patient acceptance. To address these challenges, we designed an artificial intelligence (AI)-empowered wireless video endoscopic capsule that surpasses the performance of the existing solutions by featuring, among others: (1) real-time image processing using onboard deep neural networks (DNN), (2) enhanced visualization of the mucous layer by deploying both white-light and narrow-band imaging, (3) on-the-go task modification and DNN update using over-the-air-programming and (4) bi-directional communication with patient’s personal electronic devices to report important findings. We tested our solution in an in vivo setting, by administrating our endoscopic capsule to a pig under general anesthesia. All novel features, successfully implemented on a single platform, were validated. Our study lays the groundwork for clinically implementing a new generation of endoscopic capsules, which will significantly improve early diagnosis of upper and lower GI tract diseases.

## Introduction

Gastrointestinal (GI) tract disorders are responsible for around 1 million deaths per year across Europe; and are associated with substantial morbidity and healthcare costs. The incidence and prevalence of many GI tract disorders are highest amongst the young and specially, the elderly, and as the world population ages, the disease burden will inevitably increase^1^.

Colorectal cancer (CRC) is the most common GI cancer in Europe, with a risk of CRC diagnosis in a lifetime of 1 in 20. The incidence in Europe is expected to increase from 3.6 million cases in 2015 to 4.3 million cases in 2035, due to increased life expectancy and adoption of western diet and lifestyle. Although the prognosis for early CRC diagnosis and 5-year survival can reach 70%, it stays extremely poor for late cases, being as low as 10%^1^.

Fecal immunochemical test (FIT) is routinely used in screening for CRC. If positive, patients undergo either an additional diagnostic procedure (such as colonoscopy, flexible sigmoidoscopy, computed tomographic colonography (CTC), or wireless capsule endoscopy), or a therapeutic procedure such as optical colonoscopy, to find precursors of cancer, e.g. suspicious colorectal polyps. The challenge we are facing is that only 66% of people at risk are screened comprehensively^2^. This is mainly due to the low accuracy of these screening methods, as FIT can only detect approximately between 13% to 50% of cancer with one round of screening in asymptomatic patients^3^. Low accuracy of screening methods leads to unnecessary invasive diagnostic procedures.

Diagnosing small or large bowel lesions after an onset of symptoms carries its own risks. For instance, colonoscopy only reaches the terminal ileum and is a highly specialized, technically difficult and expensive procedure, with the potential of causing serious complications including perforations, abdominal pain, and bleeding. Complete colonoscopy is not always possible due to technical difficulties (15% of cases^4^), poor bowel preparation (16.9% of cases^5^), or patient intolerance (11.1% among men and 18.4% among women^6^). Despite these potential challenges, colonoscopy remains as the gold standard for investigation of symptoms suggestive of colorectal cancer, and the only choice (besides surgery) for both diagnostic and therapeutic examinations, offering resection of polyps, if needed. CTC is an alternative, less invasive test; however, additional investigation after CTC is needed to confirm suspected colonic lesions, and this is an important factor in establishing the feasibility of CTC as an alternative to colonoscopy^7^.

Novel imaging solutions such as wireless video capsule endoscopy, hereafter referred to as camera pill, have been emerging, which offer advantages in diagnosis, follow up, and management of GI tract disorders^8^. Capsule endoscopy has been suggested as an alternative to CTC following incomplete colonoscopy. It is shown that the completion rate of CTC is slightly higher than that of capsule endoscopy (98% vs. 90%), while the diagnostic yield of capsule endoscopy was higher (37% vs. 10%), especially when comparing findings of polyps of all sizes^9^. For a thorough comparison of colonoscopy, CTC and capsule endoscopy, we refer the interested readers to the VICOCA study^10^.

While we are experiencing the winter of novel camera pills, off-the-shelf products deployed in clinical practice have not been updated for over a decade, leaving a large gap between what recent advances in embedded electronics, systems-on-chips (SOCs) and AI could potentially offer, and the solutions currently deployed in everyday practice. A thorough overview of the literature^11^ proves that a significant effort has been placed around coping challenges, such as motion control and localization^12^, navigation and manipulation of the camera pill^13^. Although the outcome of these studies and projects like VECTOR^14^ undoubtedly advanced the field, none paved the way into clinical deployment, due to bearing serious concerns on safety, size, functionality of these solutions, and technological readiness^15^.

Some of the important limitations of current off-the-shelf products include, but are not limited to: (1) logistical challenges around the delivery and collection of the camera pill, data logger and the receiver vest, (2) tedious manual diagnosis of roughly 50,000 retrieved images from each patient investigation to look for important findings, (3) low resolution images missing details as captured only under white light, (4) high incompletion rate among investigations, and 5) a more restrict bowel preparation process compared to colonoscopy or CTC^16^. Given the numerous attempts reported in the literature, with limited success towards the design of active endoscopic capsules featuring locomotion, we investigated an alternative approach, i.e., a camera pill with basic mechanical design, but empowered by disruptive on-board intelligence; which addresses the above mentioned limitations in one single platform.

The overarching aim of our study was to enhance efficiency and diagnostic accuracy of premalignant and malignant small and large bowel disorders by designing a new generation of AI-empowered camera pills. This resulted in an edge camera pill that is within limits of ingestible size, while featuring a myriad set of unprecedented functionalities towards targeted and localized small and large bowel screening and diagnostics. The most important features include: (1) onboard analysis of captured images using Deep Neural Networks (DNNs) for real-time on-the-go detection of important findings, such as bleeding, lesions, colorectal polyps or cancer, (2) image acquisition using adaptive frame rate, (3) enhanced visualization of the mucous using optical virtual chromoendoscopy (VCE) by integrating an ambient light of blue and green wavelength generated by special LEDs, mimicking both white-light-imaging (WLI) and narrow-band-imaging (NBI), (4) embedded open-source platform, where DNNs can be readily overwritten or further updated based on the task they are trained for, using over-the-air-programming (OTAP), (5) bi-directional communication between the camera pill and patient’s personal electronic devices (e.g. mobile phones or tablets) without the need of a data storage unit or additional receivers, and (6) optimal energy consumption due to the transmission of particular images with important findings.

The organization of this paper is as follows. In “System design” section, the architecture of our camera pill at component and system level, and the rationale behind its design are presented. In “Onboard intelligence” section, the details of our edge-AI solution for real-time detection of abnormalities in captured images are provided. The experimental settings in which the performance of our camera pill was tested is sketched in section “Experimental setup”. Discussions and future improvements to our design are presented in Section “Concluding remarks and future work”.

## Methods

### System design

**Figure 1:**
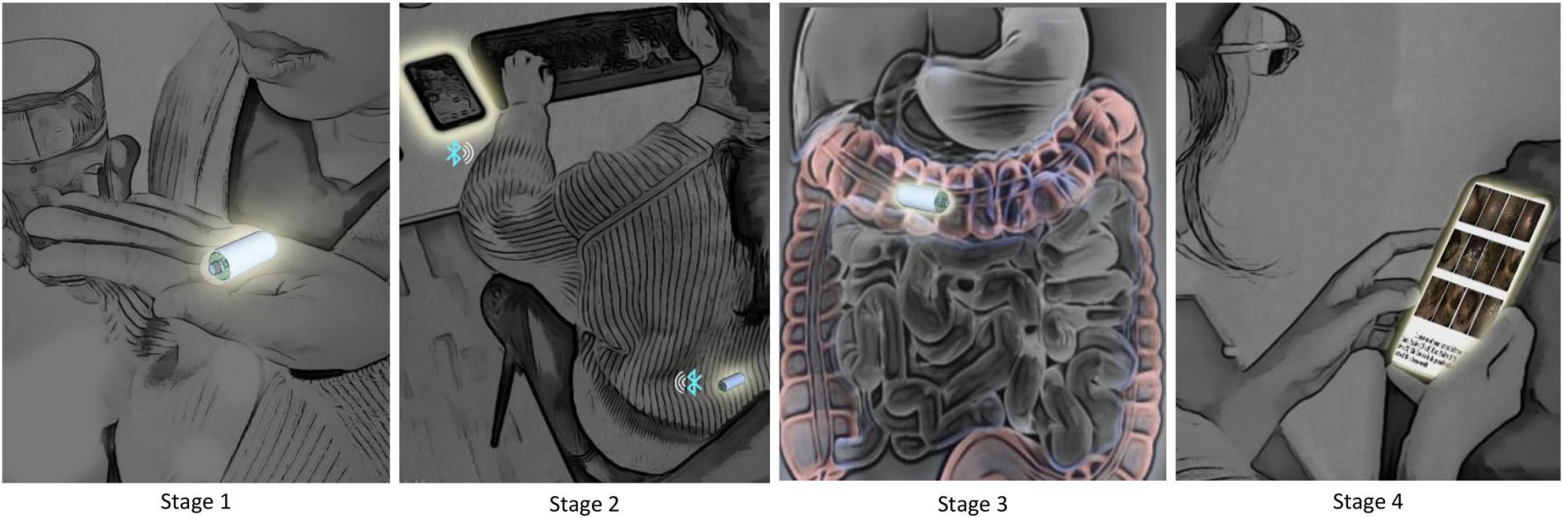
The 4 stages illustrating the use of our camera pill.

Our system design is architectured around incorporating adaptive and embedded AI, which eliminates both hardware and embedded-software barriers that impede the transition from a passive camera pill to an intelligent one. Onboard AI and OTAP are the distinct modi operandi of our camera pill compared to that of other solutions. Undergoing a UGI-LGI investigation using our camera pill, and the steps involved in the process are sketched in Fig. 1.

Once the bowel preparation procedure is complete, i.e., post-fasting and taking clear liquids, and prior to the administration of the camera pill, the pill is wirelessly programmed with pre-trained AI algorithms to detect lesions and abnormalities of interest. The onboard AI algorithms contain features such as identification of anatomical UGI and LGI landmarks for hibernation or resumption of region-specific tasks, as well as detection and localization of important lesions. After reaching the regoin-of-interest (UGI or LGI), images of the mucous layer under the while light, and at a constant frame rate of one per second are acquired, while simultaneously, onboard real-time processing of these images using pretrained DNNs are carried out. If no lesions are detected within each frame, the image will be deleted and only every 5th image will be wirelessly transmitted, so that upon completion of investigation, localization of the camera pill within UGI-LGI, and reconstruction of the bowel are carried out^17^. This was targeted towards the reduction of both unnecessary communication (bandwidth) and power consumption.

Upon detection of any abnormality such as ulcers or colorectal polyps, the frame rate is autonomously increased to up to 2fps, while real-time analysis of captured images under both white light and narrow band are carried out. All images containing important findings will be wirelessly transmitted to the receiver. After completion of investigations, image-based path reconstruction of the bowel and pinning the findings down on the reconstructed path will be performed^17^. During the investigation and after completion, all findings will be available immediately, and if a following therapeutic endoscopy or colonoscopy is recommended, the patient can go directly for an out-clinic procedure if this is available within a realistic geographical distance while benefiting from the same bowel preparation procedure deployed during camera pill investigations. In addition, the specialist will be guided to the RoI (region-of-interest) where important findings and abnormalities are pinned using the image-based reconstructed bowel.

### Hardware system architecture

**Figure 2:**
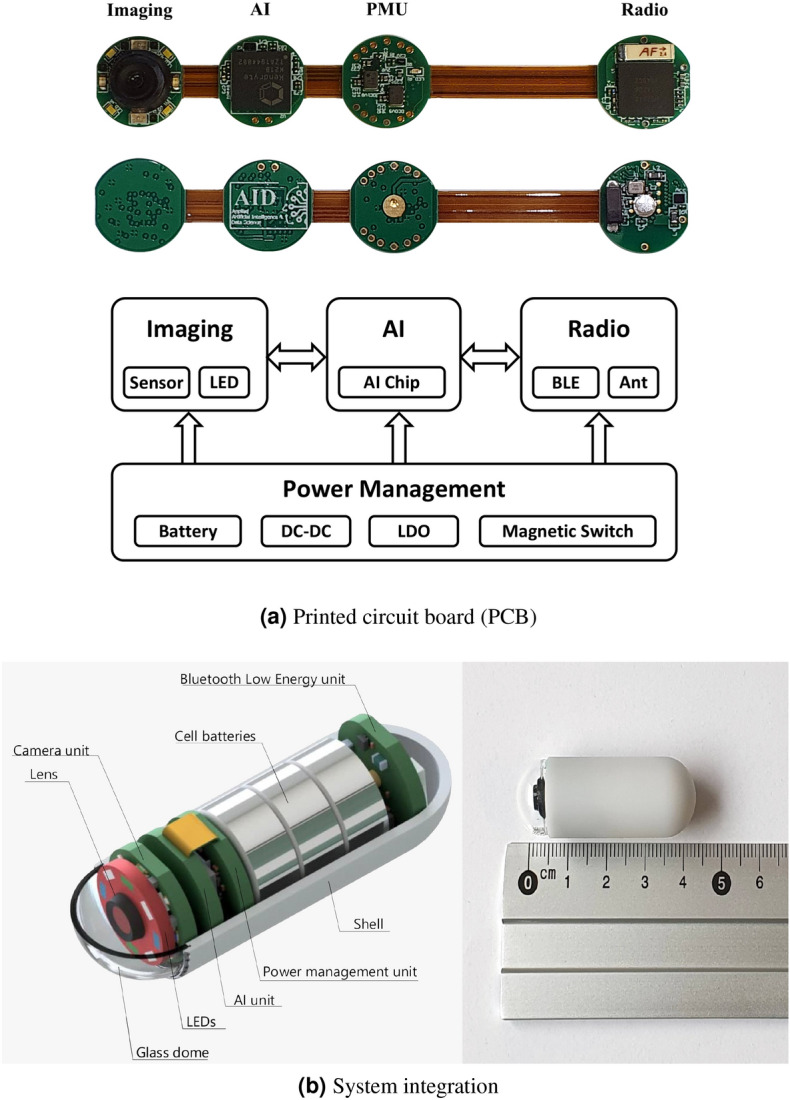
Camera pill’s PCB (**a**), system architecture of our camera pill (**b**, left), and the prototype (designed in Autodesk Inventor 2021; https://www.autodesk.com/products/inventor/overview) and sealed components (**b**, right).

System architecture and the outward appearance of our camera pill are presented in Fig. 2. The list of main components include: an AI edge computing chip, power management unit (PMU), Bluetooth low energy (BLE) bi-directional communication unit, image acquisition system (glass dome, lens, LEDs (white, green and blue) and camera unit), cell batteries, a MEMS (micro-electromechanical system) chip pack combining a 3D accelerometer and a temperature sensor, and the shell. The camera pill is 42.7 *mm* long and 15.8 *mm* wide. A side-by-side comparison of our camera pill designed in Autodesk Inventor Version 2021^18^, and a ruler can be found in Fig. 2b.

#### Image acquisition system

The image acquisition system of our camera pill consists of a glass dome (15 mm transparent small half globe glass cover) and an OV7670 CMOS VGA camera chip and lens, featuring an axial field of view of $140^{\circ }$ and a depth of field between 5 and 100mm, capturing images in BMP format with an image resolution of $240\times 240$ pixels^19^. Although a dual-headed camera pill (one camera at either end) provides a wider coverage of the mucous layer compared to a standard single-headed one, no statistically significant improvement in diagnostic yield within small bowel has been reported in the literature^20^. Without the added benefit of improving the diagnostic yield, a dual-headed camera pill compared to a single-headed counterpart can unnecessarily prolong the physician reading time and increase system’s power consumption and communication throughput. Though the diagnostic sufficiency of utilizing one camera could be linked to the small bowel’s diameter, it might not hold for large bowel investigations. Given the large diameter of colon, and the challenge of detecting laterally spreading tumors (LST) and hidden polyps, two cameras would most likely be preferred. This however entails more demanding power and computational resources. It is important to highlight that our design has the capacity to adapt to two cameras. A thorough discussion is provided in the concluding remarks and the future works of this study.

Our optical VCE Imaging modality featuring different lighting conditions, i.e., WLI and NBI, was designed by deploying a total of 8 LEDs, where the ambient light was generated by 4 white LEDs in WLI mode, and two blue and two green LEDs in the NBI mode. It is worth noting that our design is substantially different from digital VCE solutions such as the flexible spectral color enhancement (FICE), where digital post processing of videos are performed, after the image acquisition process is completed^21^.

#### AI edge computing chip

After capturing every image of the mucous layer, the images are transferred to the AI unit where onboard analysis takes place. Our camera pill is equipped with an $8~mm\times 8~mm$ K210 Kendryte chip, which is an AI Application Specific Integrated Circuit (ASIC) with the computational power of 1 tera operations per second (TOPS)^22^. K210 is equipped with 8MB of RAM, a dual core 64-bit CPU with an adjustable frequency of 400MHz. It also features KPU (neural network processor) functionalities with built-in convolution, batch normalization, activation, and pooling operations. The major advantage of K210 is the design flexibility, as there is no direct limit on the number of network layers that need to be deployed, and each layer of DNN parameters can be configured separately, including the number of input and output channels, line width and column height. In addition, K210 supports any form of activation function^22^.

#### Communication unit

Onboard analysis of captured images and solely transmitting important findings takes the edge of both communication bandwidth and power consumption. Constrained by the limited energy resources available (consisting of three cell batteries), and in order to maintain a secure direct bi-directional communication between the camera pill and patient’s personal electronic device, Bluetooth Low Energy (BLE) technology was selected. Our camera pill is equipped with a flexible single chip nRF52840 High-end multiprotocol Bluetooth 5 system-on-chip (SoC) solution featuring both long range and high throughput modes (2 Mbps), operating at 2.4 GHz ISM band^23^. The antenna solution was based on an ANT-2.45-CHP, featuring one of the smallest, low loss, high bandwidth and performance antenna chips available^24^. This choice combined with the bi-directional BLE technology enabled us to receive the images directly on a tablet without any additional interface (such as a receiver vest).

#### Power management unit

Presented in Fig. 2b, our camera pill is powered by three cell batteries, while the PMU governs various power functionalities and provides multiple voltage levels needed by different components using highly efficient DC-DC converters and linear low-dropout regulators (LDO). As the initial supplied voltage of 4.5*V* provided by the batteries drops gradually as the system operates, the PMU regulates required voltages by each system component, until the threshold of 2.8*V* is reached. Below this limit, the system no longer operates, as some of the important components such as the AI unit and the image acquisition system cannot be operational anymore.

### Onboard intelligence

Onboard DNN-based processing of captured images for detection and localization of abnormalities commonly relies on pretrained networks such as Faster-Region DNN^25^, Single-Shot-Detector (SSD)^26^ and You-Only-Look-Once (YOLO)^27–29^, among others. The main obstacle in adopting these networks for devices with limited computational resources such as our camera pill with a memory size of only 8MB, is their large size, as for instance, YOLO V3 being one of the smallest and most popular networks requires an approximate memory allocation of 237MB. In two independent studies^30,31^, we showed that ZF-Net based DNN as backbone for a Faster R-CNN to detect and localize colorectal polyps, and a ResNet-50 based convolutional neural network (CNN) to detect and classify lesions in Crohn’s disease in both small and large bowel required an approximate memory allocation of 375MB and 167MB, respectively. Keeping the constraints dictated by the memory allowance and network size in mind, we designed an optimized YOLO-based DNN of approximately 3.2MB to detect and localize colorectal polyps, and implemented the solution in our camera pill. The same concept could be applied to a variety of UGI and LGI diseases.

**Figure 3:**
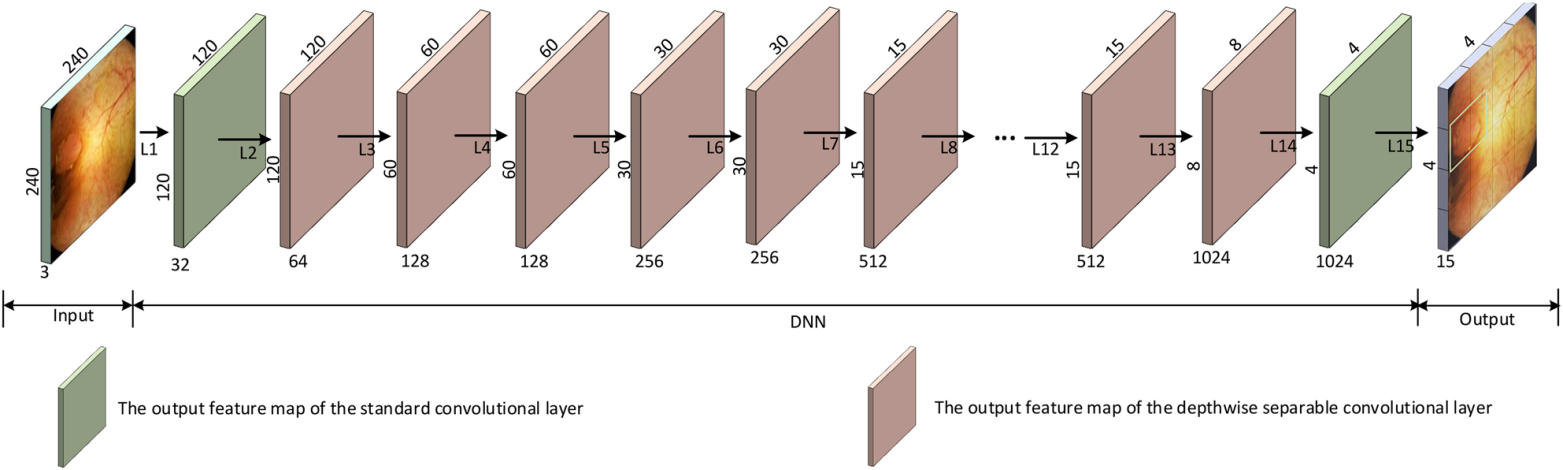
Architecture of our DNN for detection of colorectal polyps.

The architecture of our DNN is inspired by those of MobileNet^32^ and YOLO, and is based on non-maximum suppression (NMS) algorithm^33^ filtering candidate regions using a confidence score $S_p$. The algorithm then returns the location of detected colorectal polyps as a set of bounding boxes featured by the coordinates of the upper left corner [*x*, *y*], the width *w* and the height *h* of the bounding box. Images fulfilling the condition $S_p \ge S_t$ are considered to present significant findings, and are further analyzed and transferred to the BLE unit for communication to the external receiver. Images with the score not meeting the threshold will be considered insignificant, and will be deleted by the AI unit. The architecture of our proposed DNN is shown in Fig. 3.

#### DNN architecture

The architectural details of our proposed DNN for the detection and localization of colorectal polyps is presented in Table 1. Our DNN, developed in TensorFlow, has an input layer fed with images of the size $240\times 240\times 3$, 15 intermediate layers represented as $L_i$, where $i=1,\cdots ,15$, and an output layer. Two types of convolutional layers, namely standard^32^ $(L_1  \&  L_{15})$ and depth-wise separable layers^32^ $(L_2,\cdots ,L_{14})$ have been utilized. Given that the standard convolutional layers maintain useful information within an image, they were chosen as the building blocks for the first and last layer. Furthermore, to reduce the number of trainable parameters and therefore computational complexity of the network by a factor of up to $k^2$, *k* being the kernel size, depth-wise separable convolutional layers were chosen as $(L_2,\cdots ,L_{14})$ layers. These layers split the convolutional computation into two steps, 1) depth-wise convolution applying a single convolutional filter per each input channel, and 2) point-wise convolution creating a linear combination of the output of the depth-wise convolution^29^. Each convolutional layer was then followed by a batch normalization layer, which for the sake of brevity is not discussed in detail here. The shape of deployed filters are formulated as $w_k\times h_k \times c_k \times n_k$, where $w_k, h_k, c_k$ and $n_k$ are the kernel width, kernel height, number of kernel channels and the number of kernels, respectively. The stride size is set to either 1 ($S_1$) or 2 ($S_2$), while network’s input and output size are presented in the form of $w_f\times h_f \times c_f$, where $w_f,h_f$ and $c_f$ are the width, height, and channel numbers of feature maps, respectively. Our DNN outputs a total of $4 \times 4 \times 3$ proposal bounding boxes, in which after applying the NMS algorithm, the one with the largest $S_p$ is filtered as the candidate proposal. Since five elements ($S_p$, *x*, *y*, *w* and *h*) represent one bounding box, our DNN’s output size is $4 \times 4 \times 15$.

**Table 1:** On-board DNN Architecture.

Layer	Type	Filter Shape	Stride	Input Size	Output Size
L1	Conv	$3\times 3\times 3\times 32$	S2	$240\times 240\times 3$	$120\times 120\times 32$
L2	Depthwise	$3\times 3\times 32\times 1$	S1	$120\times 120\times 32$	$120\times 120\times 64$
	Pointwise	$1\times 1\times 32\times 64$	S1		
L3	Depthwise	$3\times 3\times 64\times 1$	S2	$120\times 120\times 64$	$60 \times 60 \times 128$
	Pointwise	$1\times 1\times 64\times 128$	S1		
L4	Depthwise	$3\times 3 \times 128\times 1$	S1	$60 \times 60 \times 128$	$60 \times 60 \times 128$
	Pointwise	$1 \times 1 \times 128 \times 256$	S1		
L5	Depthwise	$3 \times 3 \times 128\times 1$	S2	$60 \times 60 \times 128$	$30 \times 30 \times 256$
	Pointwise	$1 \times 1 \times 128 \times 256$	S1		
L6	Depthwise	$3 \times 3 \times 256\times 1$	S1	$30 \times 30 \times 256$	$30 \times 30 \times 256$
	Pointwise	$1 \times 1 \times 256 \times 256$	S1		
L7	Depthwise	$3 \times 3 \times 256\times 1$	S2	$30 \times 30 \times 256$	$15 \times 15 \times 512$
	Pointwise	$1 \times 1 \times 256 \times 512$	S1		
L8-L12	${\textbf {5}}\times$Depthwise	$3 \times 3 \times 512\times 1$	S1	$15 \times 15 \times 512$	$15 \times 15 \times 512$
	Pointwise	$1 \times 1 \times 512 \times 512$	S1		
L13	Depthwise	$3 \times 3 \times 512\times 1$	S2	$15 \times 15 \times 512$	$8 \times 8 \times 1024$
	Pointwise	$1 \times 1 \times 512 \times 1024$	S1		
L14	Depthwise	$3 \times 3 \times 1024\times 1$	S2	$8 \times 8 \times 1024$	$4 \times 4 \times 1024$
	Pointwise	$1 \times 1 \times 1024 \times 1024$	S1		
L15	Conv	$1 \times 1 \times 1024 \times 15$	S1	$4 \times 4 \times 1024$	$4 \times 4 \times 15$
Output					$4 \times 4 \times 15$

There is a Batch Normalization (BN) after each layer. The BNs are not shown for keeping the table concise.

#### Image acquisition and training

The images used in this study for training, validation and testing of our DNN were obtained from videos of a double blinded longitudinal trial including 255 patients from the National screening program in Denmark that were FIT-positive, during a period of one year (2015-2016). The participants underwent both colon capsule endoscopy and optical colonoscopy in two consecutive days. Prior to the day of undergoing colonoscopy, the participants were investigated by a second-generation PillCam capsule endoscopy (PillCam COLON 2 Medtronic, Minnesota, USA). Throughout both investigations, polyp size, morphology and location were identified^16^. The study was approved by the Local Ethics Committee (S20140141) and registered at clinicaltrials.gov (NCT02303756). The study was performed in accordance with relevant guidelines and regulations, and informed consent was obtained from all participants and/or their legal guardians.

**Figure 4:**
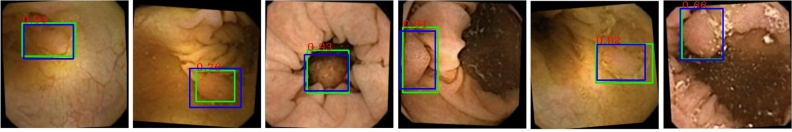
Examples of the performance of polyp detection algorithm.

The videos featuring at least one colorectal polyp of any size or morphology were analyzed frame-by-frame, which resulted in an original database of 764 distinct images of polyps. These 764 images were retrieved from 549 distinct polyps, as some of these polyps were reported as a sequence of images when the camera pill approached the polyp. The population under study was FIT positive, and approximately 75% of the polyps were neoplastic. The number of cancerous polyps was below 5%. To maintain a class balance, we further included 764 images of the normal mucous layer, where no significant pathology were detected. To regularize the network, reduce overfitting, ensure that it is rotation and translation invariant, and help remedy the scarcity of data (i.e., to increase the effective size of our dataset), we augmented the database of original images tenfold, using random rotation, scaling, translation, flipping and cropping. We also performed random mirroring at training time. The augmented images containing polyps were all checked for contents, ensuring that polyps were not mistakenly cropped out. These augmentations are justified since masses have no inherent orientation and their diagnosis is invariant to these transformations. Using data augmentation, we created a database containing 15280 images with different grades of colon cleanliness, of which half of them contained colorectal polyps of various sizes and morphology. We then split the images randomly by patient into training, validation and testing sets (80%, 10% and 10% of the full dataset, respectively), constraining the validation and test sets to be balanced. Training, validation and testing of DNN’s performance were carried out in TensorFlow, using a batch size of 20, with the training step size of $1e-3$. In addition, the NMS threshold $S_t$ was set to 0.65. A set of examples demonstrating the performance of the trained DNN is presented in Fig. 4, where blue boxes indicate the ground truth annotated manually by the specialist, and green boxes are the output of our DNN. Red numbers above each green box is DNN’s confidence value (range [0, 1]), in which larger numbers are an indication of network’s confidence in regions featuring important findings. The overall performance of our DNN for detection of colorectal polyps within the test set reached a precision of $AP_{25}=99.5\%$ and $AP_{50}=95.8\%$, $AP_n$ referring to the average precision calculated using the intersection over union (IoU) criterion.

#### DNN implementation

Our DNN features 3.2M trainable parameters, requiring a memory allocation of 3.2MB when 8-bit integer format (INT8) is used to represent each parameter. At the clock frequency rate of 400MHz for 8-bit integer computation, the power consumption of the AI chip is approximately 300mW^22^. To boost the power efficiency of the chip during the inference phase and to reduce the power required for AI computation, we reduced the clock frequency of the CPU and the AI accelerator tenfold, resulting in a power consumption of approximately 50mW. Given that voltage scaling is not a feature of K210 AI chip, the power resource of 4.5V supplies our camera pill at $0\%$ sparsity level for a time period of one hour.

### Experimental setup

**Figure 5:**
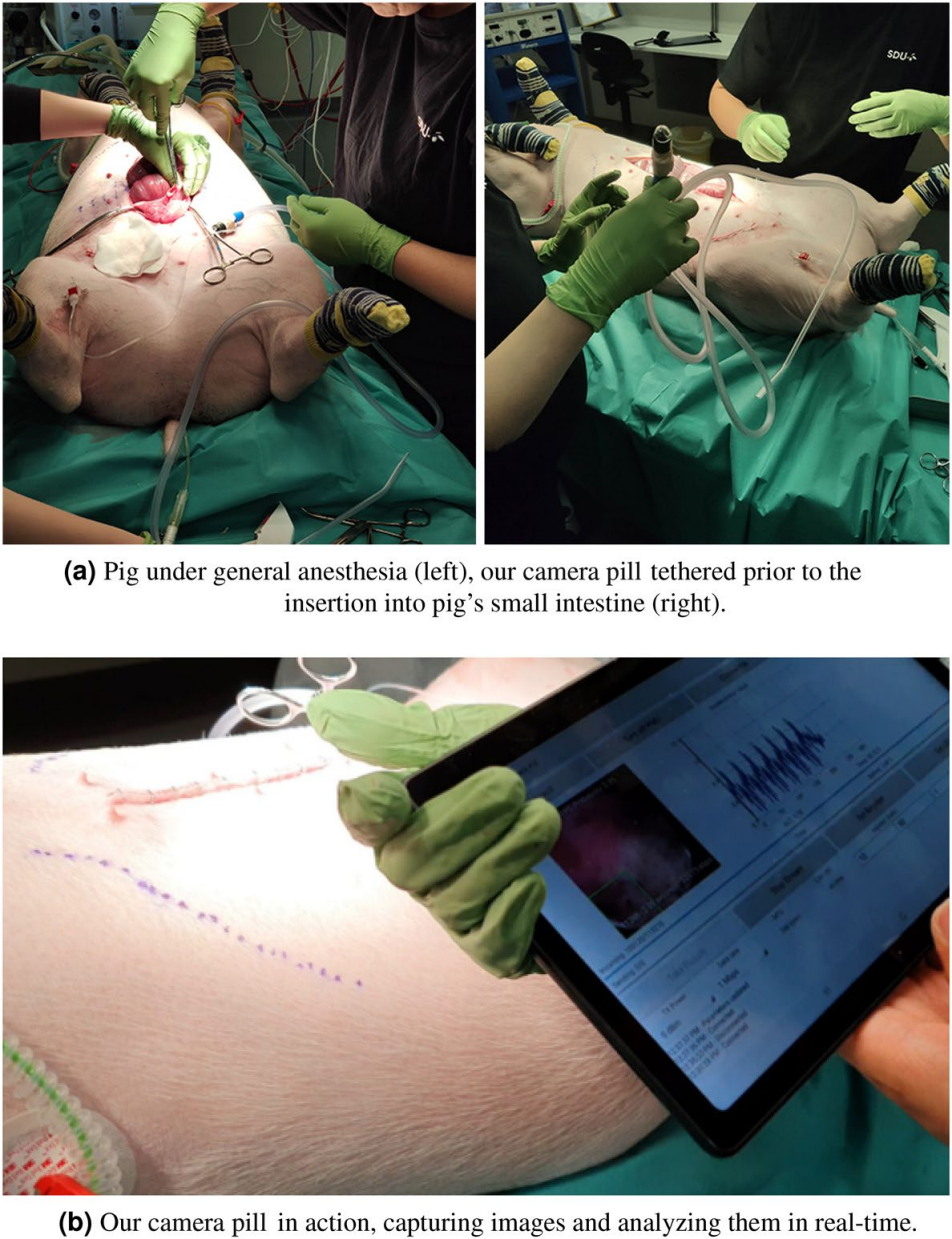
The experimental setup.

To evaluate the performance of our camera pill, and to ensure the integrity of both system design as well as individual components, we administered our camera pill to a pig under general anesthesia, as shown in Fig. 5. The pig ($\approx 45$ Kg) was intubated after premedication with sedatives (midazolam 0.2 mg/kg, medetomidin 0.024 mg/kg, ketamin 5 mg/kg and butorphanol 0.2 mg/kg IM) and ventilated with $2\%$ isoflurane in air and oxygen (1 : 2). After the procedure, the pig was euthanized while still in anaesthesia with an overdose of anaesthetics (pentobarbital 400 mg/kg IV).This study is reported in accordance with ARRIVE guidelines. The experiment was performed in accordance with relevant guidelines and regulations.

**Figure 6:**
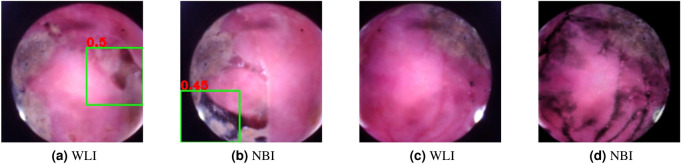
White light (WLI) and narrow band imaging (NBI) modalities.

To avoid retention, the camera pill was anchored to the tip of a flexible tube, and then inserted through the abdominal wall into the small intestine. The abdominal wall was then sutured and the tethered camera pill was guided to different locations through the small intestine, where the performance of the MEMS chip pack (temperature and acceleration) was tested and validated. Prior to the insertion, we over-the-air programmed the camera pill with the DNN trained for detecting colorectal polyps. It can be observed in Fig. 6a and b, that the DNN detects regions that appear as haemorrhagic (red color) areas and localizes them as colorectal polyps. Once the collection of several images of the mucous layer under WLI modality was completed, and corresponding bounding boxes were calculated, we switched to the NBI mode. An example of the mucous layer seen under both modalities are presented in Fig. 6c and d, exhibiting an enhanced contrast and elevated level of details during NBI compared to that of WLI. To test the functionality of the camera pill inside pig’s intestine and to verify the performance of the BLE unit, AI component and the communication between these two entities, we designed a mock-up of a similar DNN to the one sketched previously, but featuring different values for network’s parameters. We then uploaded the mock-up DNN using OTAP from a tablet, and successfully rewrote the existing network that was trained for detecting colorectal polyps, while the camera pill was inside pig’s small intestine. The experiment was carried out for approximately 1-hour at 0% sparsity state, until the cell batteries were depleted. This is the state in which the camera pill was 100% in active mode, detecting polyps, performing semantic segmentation, and wirelessly transmitting the images. This is equivalent to a scenario in which every image captured by the camera pill featured an important finding. It is evident that by increasing the sparsity level as occurring during patient investigation, the camera pill will operate for longer periods of time, fulfilling $8-10$ hours of recording. The data collected during the experiment were saved, and the entire experiment was recorded by two digital cameras from different angles.

#### Protocol approval

The study (double-blinded longitudinal trial) was approved by the Danish Local Ethics Committee (S20140141) and registered at clinicaltrials.gov (NCT02303756) on December 1st 2014^34^. All participants were informed about the study, and signed, written informed consents to publish the results of this study were obtained. Furthermore, informed consent for participation was obtained from the participants in the manuscript. For a more detailed description of the study design, we refer the interested readers to the supplementary materials and our previous publications^6,8,16^.

The in vivo procedure that was carried out on the pig, was ethically approved by the Danish Animal Inspectorate under license 2016-15-0201-00815. Animal housing, care and preparation were performed by qualified staff under the supervision of a designated veterinarian at the Animal Medicine Laboratory, Medical Research Center at the University of Southern Denmark, with the approval of the institutional animal welfare body (AWB), the equivalent of the Institutional Animal Care and Use Committee (IACUC).

## Concluding remarks and future work

Our camera pill, featuring onboard intelligence, successfully addresses major limitations of current off-the-shelf products deployed in clinical practice. The design of our camera pill, capable of providing both predictive and reactive intelligence, based on the collected images, is feasible, mainly due to recent advances in deep learning and surge of interest in embedded AI. Features such as onboard real-time processing of images for lesion detection and classification, bi-directional communication (OTAP) to modify assigned tasks on-the-go, and improved image resolution and enhanced quality by means of optical chromoendoscopy, all included on a single platform, can tip the scales in favor of the novel camera pill design presented here, potentially making it the “device-of-choice” for both CRC screening and diagnostic purposes. Furthermore, issues such as poor bowel preparation, incomplete investigation, and exorbitant costs of logistics around the delivery of the camera pill, followed by retrieving the equipment and reader’s investigation sessions, inter alia, that hinder universal acceptance of capsule endoscopy can be resolved by using our design.

Based on the results, it is evident that the addition of both blue and green wavelength by deploying extra LEDs, to mimic the spectrum procured during NBI resulted in a remarkable improvement in image quality, which in turn has the potential to significantly reduce the need for intensive *a priori* cleansing regimen of the GI tract, and for a strict patient preparation process. The use of NBI further provides enhanced vessel and surface patterns of lesions that contribute to both detection and characterization of those lesions. Despite the fact that our DNN was trained to detect and localize colorectal polyps, the in vivo evaluation of its performance inside the small intestine of a pig was satisfactory, as regions appearing to contain lesions similar to polyps were proposed by the network, albeit justifiably with low confidence.

Our future efforts will focus on addressing the shortcomings of our current design, and the evaluation process around it. Reducing the size, primarily the length of our camera pill, is of paramount importance. Our design is approximately 1cm longer than commercially available products such as Colon2 PillCam ($11.6~mm\times 32.8~mm$) or SB3 PillCam ($11.4~mm\times 26.2~mm$), which can be pinned on the use of three cell batteries. It is worth mentioning that the main challenge we faced during the design process of our camera pill to make it comparable to its counterparts (Medtronic PillCam or IntroMedic MiroCam) in terms of size, image resolution and the field of view, was the inaccessibility of the components to be purchased for research purposes (i.e., small scale purchase). Several components that we integrated in our solution, such as the imaging system with a limited $140^{\circ }$ field of view or the AI chip with 8MB of RAM were the most advanced units. This obstacle, to a large degree, dictated us the choice of components. At the time of conducting the study, K210 Kendryte chip and the OV7670 CMOS VGA camera chip and lens were the only viable choices, dictating the needs for such power resources. This limitation, however, does not hinder the functionality of our camera pill, which could be readily improved by adopting more advanced components. This will be addressed in our next design, as computationally more powerful and less power demanding AI chips are now available, enabling us to train more computationally complex and larger DNNs. Our current DNN features an input layer targeting images of size $240\times 240$ pixels, which is approximately half the number of pixels retrieved from Medtronic PillCams ($340\times 340$ pixels). Combined with the double-headed camera design, Medtronic PillCams produce four times the number of pixels retrieved from our solution. Large number of pixels have a significant impact on the processing time, communication throughput and energy resources on an embedded AI platform with limited computational and power resources as camera pills.

On the algorithmic side, we will improve our DNN to include various types of lesions, such as bleeding, or inflammatory bowel diseases. After pruning and optimization, our multi-lesion classifier based on a ResNet-50 CNN^31^ will be implemented onboard, enabling us to detect and classify lesions in Crohn’s disease in both small and large bowel. Our DNN will also identify important anatomical landmarks (e.g., flexures), and we will replace the mock-up DNN with pruned networks trained for detecting a variety of lesions, e.g., bleeding of the GI tract. Publicly available high quality annotated wireless capsule endoscopy (WCE) images and videos such as KID^35^ play a fundamental role in this journey. The next step towards the evaluation process is the administration of our camera pill to a pig, while freely roaming in a pen.

Even taking into account the few limitations of our design, our edge AI camera pill signifies an improvement of utmost importance over current camera pill solutions. Addressing challenges around the delivery and retrievement of the equipment (data logger and the receiver vest) can lead to the adoption of our solution into general practitioner’s office rather than highly specialized hospitals. In addition, mitigating the psychological burden on patients by reporting the outcome of the investigations immediately, play a major role in the acceptance of our solution by both patients and healthcare professionals, as shown in a recent patient reported outcome method (PROM) study^36^. The study showed that the current waiting time of 3 days for the patients to receive the reports on their capsule endoscopy investigation poses a major psychological burden. In a scenario where the camera pill investigation indicates important findings, recommended immediate therapeutic follow-up endoscopy or colonoscopy is feasible, since the patient’s physical condition is considered acceptable for an out-clinic colonoscopy in sedation, except those prescribed with special anticoagulants. This practice has already been planned at the Department of Surgery at the Odense University Hospital, where camera pills are administered in groups of $5-10$ patients, and 2 colonoscopy slots are reserved at the clinic.

Finally, ethical issues related to physician’s acceptance and patient’s confidence in novel computer-aided algorithms such as onboard AI needs to be discussed. Even though studies such as PEACE^37^ clearly indicate that there is a significant tendency towards AI acting as adversarial rather than assistive tool, (i.e., only selected images will be proofread by the expert, while others will simply not be double-checked), the fact that not all the images are available for a full screening process could be a cause for concern. Our platform, using OTAP, is capable of transmitting all images, if pre-indications on various lesions exist. In addition, we can adjust our DNN to obtain both a high positive predictive value (PPV) and a high negative predictive value (NPV), and therefore wirelessly transmit an increased number of images as important findings for further investigation.

## Data Availability

The data supporting the results reported in this article can be provided to interested readers, by contacting the corresponding author. However, due to the absence of consent for publication or complete anonymisation of the outcome of the clinical trial, enquiries about access to the outcome of the trial, i.e., database containing patient information and images of lesions should be made to the data owner, i.e., Odense Univesity Horpital (Svendborg Hospital).
